# AllergoOncology: Expression platform development and functional profiling of an anti‐HER2 IgE antibody

**DOI:** 10.1111/all.13818

**Published:** 2019-05-27

**Authors:** Kristina M. Ilieva, Judit Fazekas‐Singer, Heather J. Bax, Silvia Crescioli, Laura Montero‐Morales, Silvia Mele, Heng Sheng Sow, Chara Stavraka, Debra H. Josephs, James F. Spicer, Herta Steinkellner, Erika Jensen‐Jarolim, Andrew N. J. Tutt, Sophia N. Karagiannis

**Affiliations:** ^1^ Breast Cancer Now Research Unit, School of Cancer & Pharmaceutical Sciences King's College London, Guy's Cancer Centre London UK; ^2^ St. John's Institute of Dermatology, School of Basic & Medical Biosciences King's College London, Guy's Hospital London UK; ^3^ Institute of Pathophysiology and Allergy Research Medical University of Vienna Vienna Austria; ^4^ The Interuniversity Messerli Research Institute of the University of Veterinary Medicine Vienna Medical University Vienna and University Vienna Vienna Austria; ^5^ Department of Applied Genetics and Cell Biology University of Natural Resources and Life Sciences Vienna Austria; ^6^ School of Cancer & Pharmaceutical Sciences King's College London, Guy's Hopsital London UK; ^7^ Breast Cancer Now Toby Robins Research Centre Institute of Cancer Research London UK

To the Editor,

Monoclonal antibodies approved for the treatment of cancer belong to the IgG class (most often IgG1). However, IgG has limited tissue half‐life (2‐3 days), relatively low affinity for cognate Fc receptors and the disadvantage of interaction with inhibitory Fcγ receptors, abundant in the tumour microenvironment. Conversely, IgE class antibodies may offer new options for cancer therapy, based on high affinity for cognate Fcε receptors expressed on different, often tumour‐resident, immune effector cells such as macrophages and mast cells, and lack of inhibitory Fc receptors.^1^ IgE‐mediated tissue surveillance functions known to potentiate “allergic” or “pathogen/parasite‐clearing” immunity could be re‐directed against tissue‐resident tumours.^2^, ^3^ IgE antibodies recognizing the tumour‐associated antigen folate receptor α (FRα) induced superior immune responses in disparate in vivo models, highlighting potential opportunities for FRα‐expressing ovarian carcinomas.^2^ In breast cancer, in vitro studies of trastuzumab (IgG1) and an engineered trastuzumab IgE recognizing the tumour‐associated antigen HER2/*neu* indicated that IgE could complement or possibly improve the clinical performance of trastuzumab.^4^ The first‐in‐class IgE antibody (MOv18) is undergoing an early phase clinical trial in patients with FRα‐expressing carcinomas (NCT02546921, http://www.clinicaltrials.gov).

Despite considerable progress, production of monoclonal antibodies remains time‐consuming and labour‐intensive. One reason is the requirement for expression of heavy (HC) and light chains (LC) in a controlled manner, usually cloned in separate expression vectors using enzymatic restriction digestion and ligation. This introduces experimental variability in expression procedures and is often inefficient. These limitations also concern the study of anti‐allergen IgE, where Fabs rather than full‐length antibodies are commonly expressed and evaluated.^5^, ^6^, ^7^ Therefore, antibody cloning systems are moving towards utilization of single dual‐expression plasmids (eg pcDNA3.3 and pVitro1 hygro‐mcs), to increase antibody production.^8^

Building upon ours and others' previous methodologies, we report the efficient transient expression and functional evaluation of IgE, exemplified using the variable region sequences of trastuzumab and human IgE constant regions (anti‐HER2 IgE).

We employed polymerase incomplete primer extension (PIPE) PCR cloning and enzyme‐free assembly of DNA fragments. The amino acid sequences of trastuzumab variable light (VL) and heavy (VH) chain regions were manually codon‐optimized for a human expression host and cloned into a pVitro1‐hygro‐mcs dual‐expression vector containing precloned cassettes of the human epsilon HC and kappa LC using PIPE PCR cloning methodology (Figure 1A).^8^ PIPE PCR was performed using the pVitro1 plasmid to generate linear PCR fragments with 5′ PIPE overhangs, and trastuzumab variable region fragments to derive VL and VH region fragments with 5′ PIPE overhangs (DNA fragment sizes by agarose gel electrophoresis, Figure 1B).

**Figure 1:**
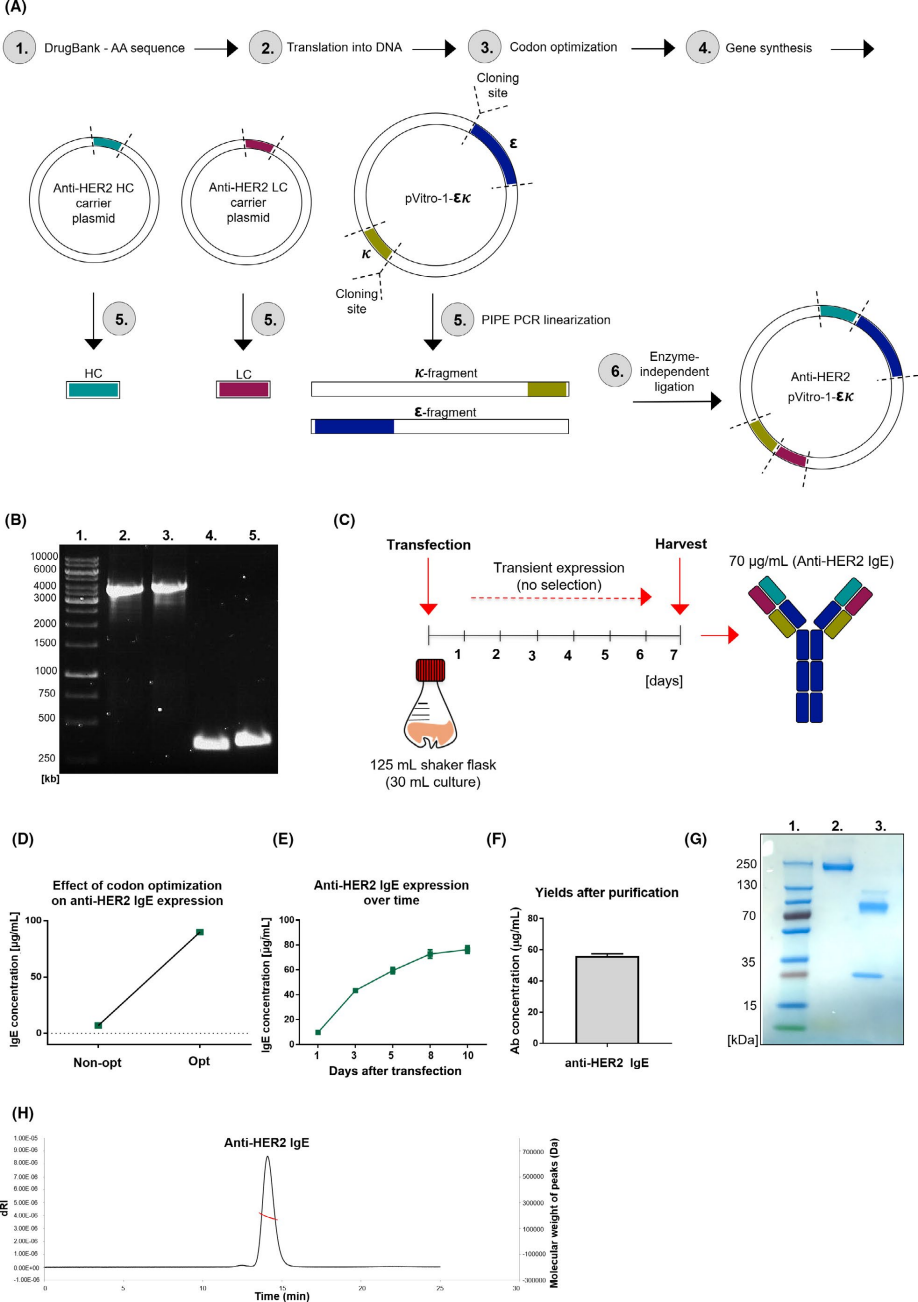
Anti‐HER2 IgE cloning and generation. A, Cloning strategy. 1‐4: Variable region DNA sequence generation. 5: Trastuzumab variable region plasmids, pVitro1 plasmid with kappa/epsilon constant chains linearized (PIPE PCR), generating 4 fragments with 5′ PIPE overhangs. 6: Linear fragments assembled nonenzymatically (pVitro‐1‐ε*κ*). B, Agarose gel electrophoresis (PIPE fragments). 1: DNA ladder, 2: ε‐fragment (4099 bp), 3: *κ*‐fragment (4119 bp), 4: LC (364 bp), 5: HC (408 bp). C, Expression strategy. D, 7‐day yields following codon optimization (representative). Expression before (E) and after (F) purification (±SD, representative of n = 2). G, SDS‐PAGE: 1: protein standard, 2: nonreducing, 3: reducing conditions. H, HPLC trace after size exclusion chromatography

Expression was conducted transiently in human embryonic kidney (Expi293F) cells without antibiotic selection, in 30 mL serum‐free suspension cultures (Figure 1C). Variable region codon optimization enhanced antibody yields (~7‐fold; Figure 1D). Peak antibody concentrations (70‐80 µg/mL) were achieved within 7‐9 days (supernatants harvested after 7 days, Figure 1C,E). After purification, total yields were 60 µg/mL (>85% purification efficiencies; Figure 1F). SDS‐PAGE of purified antibodies under nonreducing conditions showed a 250 kDa band, likely reflecting high antibody glycosylation), and reducing conditions revealed two signals (75 kDa [HC], 25 kDa [LC]), and a slight signal (100 kDa) likely representing different HC glycoforms (Figure 1G). HPLC analysis demonstrated assembly of monomeric IgE (Figure 1H).

Like trastuzumab, anti‐HER2 IgE recognized HER2/*neu*‐overexpressing (BT‐474, ZR75‐30) breast cancer cells and moderately expressing MCF‐10 normal breast cells, and its HER2 antigen recognition kinetic profile on tumour cells was comparable to trastuzumab (Figure 2A). Anti‐HER2 IgE and trastuzumab similarly restricted breast cancer cell viability and epidermal growth factor signalling, while addition of antibodies together did not improve HER2 signalling inhibition (Figure 2B,C). Consistent with FcεR‐binding MOv18 IgE^1^, ^2^ (produced in SP2/0 cells), anti‐HER2 IgE recognized RBL SX‐38 rat basophilic leukaemia cells, expressing the human tetrameric FcεRI(αβγ2), and human U937 monocytes expressing the low‐affinity IgE receptor FcεRII/CD23 upon IL‐4 stimulation (Figure 2D). Similar to MOv18 IgE, anti‐HER2 IgE recognized FcεRI‐expressing human primary monocytes and anti‐HER2 IgE binding kinetics to RBL SX‐38 were comparable to those of MOv18 IgE (Figure 2).

**Figure 2:**
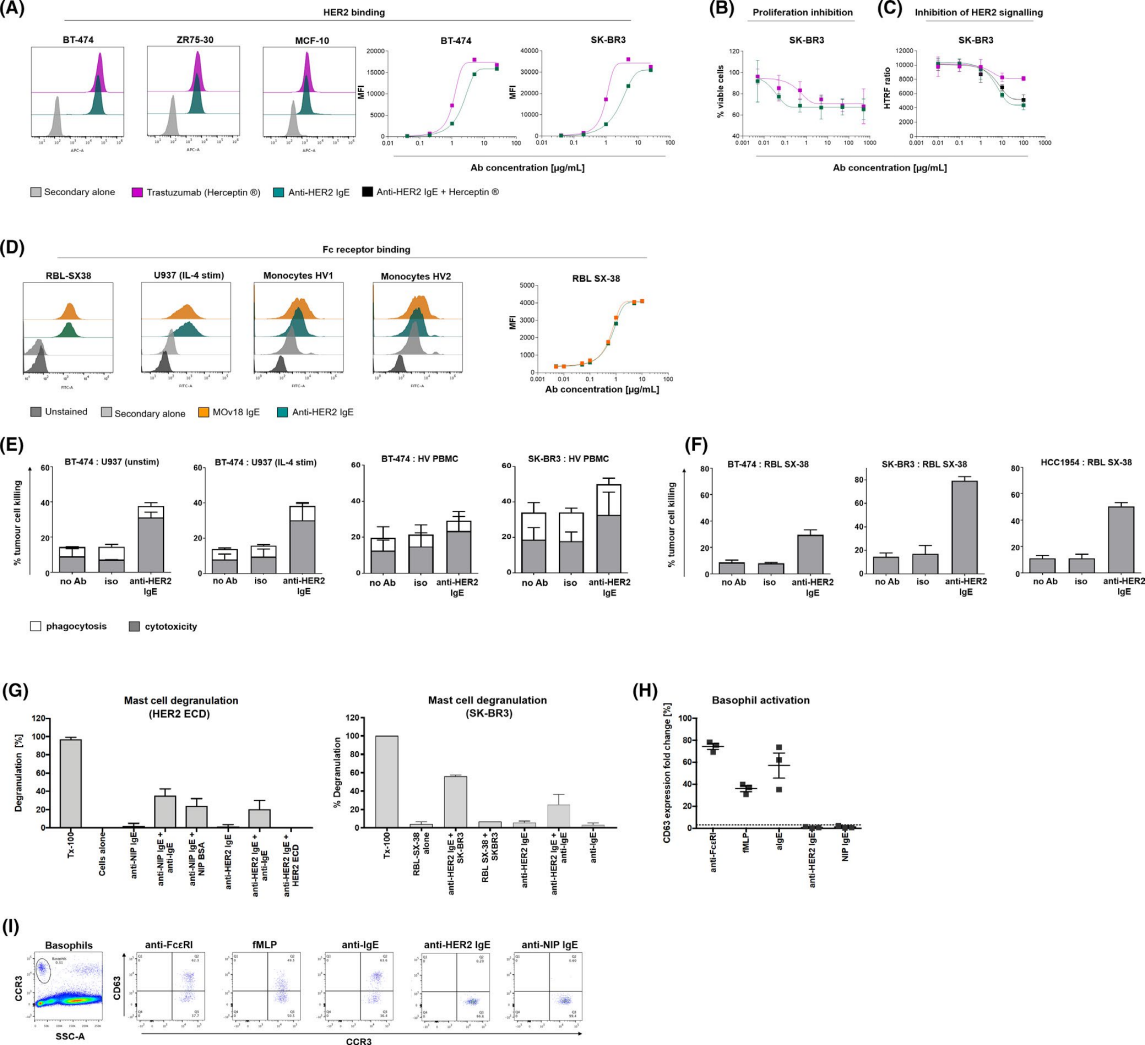
Anti‐HER2 IgE functional characterization. A, Flow cytometric binding/kinetic profiles to breast cancer and normal breast (MCF‐10) cells. IgE reduced breast cancer cell viability (B), and HER2/*neu* signalling (n = 2) (C). D, Flow cytometric binding/kinetic profiles of IgE to human FcεR‐expressing: RBL SX‐38 mast cells, U937 monocytes, human monocytes (healthy volunteers, HV; anti‐FRα IgE [MOv18]: control). E, F, IgE‐mediated % tumour cell killing (±SD): (E) by U937 (n = 3), human (HV) PBMC (n = 6); (F) by RBL SX‐38. G, RBL SX‐38 degranulation experiments (*β*‐hexosaminidase release, Triton X‐100 lysis (Tx100): 100% granule release, representative of n = 2). H, I, Anti‐HER2 IgE stimulation in basophil activation test (BAT) (G), and representative flow cytometric dot plots (I), depicting lack of basophil activation with anti‐HER2 IgE stimulation

Anti‐HER2 IgE induced >2‐fold higher ADCC of HER2‐overexpressing breast cancer cells by unstimulated and IL‐4 stimulated U937 effector cells compared with isotype controls (Figure 2E). Anti‐HER2 IgE triggered higher ADCC against breast cancer cells by peripheral blood mononuclear cells (PBMCs from human volunteers, HV, Figure 2E) and >‐fold higher ADCC by RBL SX‐38 cells (Figure 2F) compared with isotype controls (see Appendix S1).

Anti‐HER2 IgE induced degranulation of RBL SX‐38 cells when cross‐linked by polyclonal anti‐IgE on the cell surface (left) or by HER2‐expressing tumour cells (right), but not without cross‐linking stimulus or with recombinant monomeric antigen (HER2 ectodomain [ECD]; Figure 2G). In basophil activation tests (BAT) conducted in unfractionated human blood, anti‐HER2 IgE did not induce basophil activation, monitored by upregulation of the activation marker CD63 (Figure 2H,I). Mast cell and basophil tests therefore confirm lack of activation with IgE in the absence of cross‐linking stimuli,^9^ supporting potential safe administration in human circulation.

IgE immunotherapy may offer a promising approach for cancer treatment, contributing to the emerging field of AllergoOncology, focused on dissecting interplay between IgE, allergy and malignancy. The development of efficient platforms for speedy generation of full‐length IgE at appreciable yields for numerous evaluations to expedite the field remains challenging. Our herein‐described multi‐gene cloning, enzyme‐free assembly system for rapid expression of functionally active antibody, within 7‐9 days from transfection to purification in serum‐free cultures (2 mg purified material from 30 mL), readily established even in “small” environments, surpassing previous platforms in expression efficiency, speed (7‐9 days vs 4‐6 weeks) and yields (70‐80 mg/mL vs <20‐25 mg/mL),^4^ meets these challenges. IgE maintained Fab‐ and Fc‐mediated properties, including antigen and receptor binding, ADCC and degranulation, contributing to the most important/prominent antibody functionalities. These suggest that under conditions akin to those of tumours, when encountering high levels of HER2‐expressing cancer cells, anti‐HER2 IgE may trigger mast cell activation and antitumour effector functions. Importantly, the lack of anti‐HER2 IgE blood basophil activation points to diminishing potential safety concerns associated with using IgE class antibodies in cancer immunotherapy. Our report of transient cloning and rapid antibody production greatly facilitates the study of IgE structural and immune functional attributes and may find numerous applications in allergy, biotechnology and immunology‐related fields.

## CONFLICTS OF INTEREST

SN Karagiannis and JF Spicer are founders and shareholders of IGEM Therapeutics Ltd. SN Karagiannis holds a patent on antitumour IgE antibodies. All other authors have declared that no conflict of interest exists.

## Funding information

The study is a result of a collaborative effort from the AllergoOncology Task Force of the European Academy of Allergy and Clinical Immunology (EAACI). The authors acknowledge support by Breast Cancer Now (147), working in partnership with Walk the Walk; Cancer Research UK (C30122/A11527; C30122/A15774); the Medical Research Council (MR/L023091/1); the Academy of Medical Sciences; CR UK//NIHR in England/DoH for Scotland, Wales and Northern Ireland Experimental Cancer Medicine Centre (C10355/A15587). The research was supported by the National Institute for Health Research (NIHR) Biomedical Research Centre (BRC) based at Guy's and St Thomas' NHS Foundation Trust and King's College London (IS‐BRC‐1215‐20006). Further funding: Austrian Science Fund in the frame of the Doctoral Program BioToP (Grant W 1224) (L.M‐M, HS). JF‐S and EJ‐J were supported by the Austrian Science Fund, projects W1205‐B09 (doctoral college CCHD) and in part SFB F4606‐B28. JF‐S is a recipient of an EAACI (European Academy of Allergy and Clinical Immunology) Exchange Research Fellowship 2016 and a Boehringer Ingelheim Fonds Travel Grant 2017. The authors are solely responsible for study design, data collection, analysis, decision to publish and preparation of the manuscript. The views expressed are those of the author(s) and not necessarily those of the NHS, the NIHR or the Department of Health.
