# Supplementary material for: AllergoOncology: Expression platform development and functional profiling of an anti‐HER2 IgE antibody
Source: Allergy. 2019 May 27;74(10):1985–9. doi: 10.1111/all.13818 (PMC6817356; doi:10.1111/all.13818)
Supplement: Supplementary file 1 [file ALL-74-1985-s001.docx]

**Supplementary Materials and Methods**

**AllergoOncology: Expression platform development and functional profiling of an anti-HER2 IgE antibody**

Kristina M Ilieva, Judit Fazekas-Singer, Heather J Bax, Silvia Crescioli, Laura Montero-Morales, Silvia Mele, Heng Sheng Sow, Chara Stavraka, Debra H Josephs, James F Spicer, Herta Steinkellner, Erika Jensen-Jarolim, Andrew N J Tutt, Sophia N Karagiannis

**Generation and expression of anti-HER2 IgE**

Antibody cloning: We employed polymerase incomplete primer extension (PIPE) PCR cloning and enzyme-free assembly of DNA fragments. The amino acid sequences of the trastuzumab heavy and light variable regions were obtained from the DrugBank database (www.drugbank.ca), translated in nucleotide sequences and manually codon optimized for a human expression host. Optimized sequences were synthesized using GeneArt Gene Synthesis (Thermo Fischer Scientific UK). The DNA sequences of the variable regions were previously reported^1^. The variable region fragments of trastuzumab were cloned into pVitro1-hygro-mcs dual expression vector containing pre-cloned cassettes of the human epsilon chain constant region and kappa light chain constant region cassettes using the polymerase incomplete primer extension (PIPE) PCR cloning as previously described^2^. The PIPE PCR primers used for the fragment amplification are listed in Supplementary Table S1. Briefly, PIPE PCR was performed using pVitro1 plasmid as a template in order to generate two linear PCR fragments with 5` PIPE overhangs, and commercially-generated trastuzumab variable region fragments, in order to generate a variable light (VL) and heavy (VH) region fragments with 5` PIPE overhangs. Correct sizes of the DNA fragments were confirmed with agarose electrophoresis. Anti-HER2 IgE expression was carried out transiently in Expi293F cells using small volume suspension culture (30 mL) for ca. 7-9 days to achieve peak antibody concentrations of ca. 70-80 µg/mL in the culture supernatant.

Purification and HPLC: The antibody was purified using KappaSelect affinity chromatography (GE Healthcare), concentrated with Amicon centrifugal filters, (MWCO 10,000 kDa, Merck Millipore, Germany) and analysed using sodium dodecyl sulphate polyacrylamide gel electrophoresis (SDS-PAGE) using pre-cast 4-16% gels (Bio-Rad). Before performing analytical high-performance liquid chromatography (HPLC), HER2-IgE was subjected to size-exclusion chromatography (SEC) on a HiLoad 16/600 Superdex 200 pg column (GE Healthcare, USA) equilibrated with PBS plus 200 mM NaCl, and fractions pooled and concentrated. HPLC SEC combined with multi-angle light scattering was performed to confirm the molar mass of the monomeric IgE collected resulting from preparative SEC. HPLC (Shimadzu prominence LC20, Japan) was equipped with MALS (WYATT Heleos Dawn8+ plus QELS; software ASTRA 6), refractive index detector (RID-10A, Shimadzu) and a diode array detector (SPD-M20A, Shimadzu). The column (Superdex 200 10/300 GL, GE Healthcare, USA) was equilibrated with PBS plus 200 mM NaCl (pH 7.4) as running buffer. Prior to analysis, the IgE sample was centrifuged (17,000 g, 10 min, 20^0^C) and filtered (0.1 mm Ultrafree-MC filter, Merck Millipore, Germany). Experiments were performed at a flow rate of 0.75 mL min-1 at 25^0^C. The proper performance of molar mass calculation by MALS was verified by the determination of a sample of bovine serum albumin.

**Isolation of Human Immune Cells**

Human samples were collected with informed written consent, in accordance with the Helsinki Declaration. Study design was approved by the Guy’s Research Ethics Committee (REC No 07/H0804/131), Guy’s and St. Thomas’ NHS Foundation Trust. Peripheral blood was also obtained through the UK National Health System (NHS) Blood and Transplant system from anonymous donor leukocyte cones. Human peripheral blood mononuclear cells (PBMCs) were isolated using Ficoll® Paque PLUS (GE Healthcare) density gradient centrifugation. Red blood cells were lysed from extracted PBMCs using RBC Lysis buffer (Biolegend).

**Flow cytometric evaluations**

Adherent cell lines were detached using 0.25% trypsin-EDTA (Thermo Fisher Scientific UK). Human healthy volunteer peripheral blood mononuclear cells were extracted using Ficoll-Paque PLUS (GE Healthcare) density centrifugation. Cells were re-suspended in 2% foetal bovine serum in phosphate buffered saline (FACS buffer) and incubated in 96-well round-bottom plates for 30 min. The cells were washed and incubated with secondary Alexa Fluor 647 (AF647)-conjugated anti-human kappa chain antibody (Southern Biotech, UK) or FITC-conjugated goat anti-human IgE (Vector Laboratories, UK). All flow cytometric evaluations were conducted on a BD FACSCanto^TM^ II or BD LSRFortessa^TM^ cell analysers (BD Biosciences, UK). The data were analysed using FlowJo software version 10.

**Antibody-dependent cellular cytotoxicity/phagocytosis (ADCC/ADCP) assay with primary human PBMC effector cells, U937 effector cells**

ADCC/ADCP was quantified using a three-colour flow cytometric assay, modified from previously described methods^3,4^. Briefly, BT-474 and SK-BR3 breast cancer cells were pre-stained with CFSE cell tracking dye (Life Technologies) 16 hours before the assay. Next, the cancer cells were pre-incubated with 10 μg/mL anti-HER2 IgE or isotype control anti-NIP 228 IgE for 30 min at 37^o^ C, washed and mixed with freshly isolated human PBMCs at an E:T (effector to target) ratio ca. 20:1 or U937 monocytic cells (non-stimulated or pre-stimulated with IL-4) (selected from conditions tested, see Supplementary Figure S1) at E:T 3:1 in RPMI GlutaMAX^TM^ (Thermo Fischer Scientific) containing 2% FCS (Thermo Fischer Scientific). The cells were co-incubated in 5 mL test tubes (BD Biosciences) for 3 hours at 37^o^ C, 5% CO_2_. After the incubation, the primary monocytes/U937 cells were stained with anti-CD89 APC-conjugated antibody (BioLegend) on ice for 30 min. Dead cells were identified using DAPI live/dead dye (Life Technologies). The flow cytometric analyses were performed on a BD FACSCanto^TM^ and data were analysed by evaluating two colour dot plots as previously described^3,4^ (examples in Supplementary Figure S2) using the FlowJo software version 10.

**Antibody-dependent cellular cytotoxicity assay with RBL SX-38 effector cells**

Rat basophilic leukaemia RBL SX-38, stably expressing the human tetrameric (αβγ2) high-affinity IgE receptor, FcεRI, were used as effector cells in a cytotoxicity (ADCC) assay. For experiments targeting HCC1954 and SK-BR3 breast cancer cells, target cells were stained with CFSE cell tracking dye (FITC channel) 16 hours before the assay. Next, the target cells were pre-incubated with 0.5µg IgE per test for 30 min at 37^0^C and subsequently incubated with RBL SX-38 effector cells (E:T 10:1) for 2 hours at 37^0^C. BT-474 breast cancer cells were labelled with CellTrace™ Far Red (Thermo Fisher, APC channel) 16 hours prior the experiment. On the day of the experiment, the target cells were pre-incubated with 1µg IgE per test for 30 min at 37^0^C and subsequently co-cultured with RBL SX-38 effector cells (E:T 5:1) for 3.5 hours at 37^0^C. RBL SX-38 cells were detected using an anti-CD63 antibody and a secondary anti-mouse AlFl488 antibody. DAPI was added immediately before acquisition on a BD FACSCanto^TM^.

**Mast cell degranulation**

Rat basophilic leukaemia RBL SX-38 cells were used in the mast cell degranulation assays to measure β-hexosaminidase release, as previously described^5^. Controls were: unstimulated cells; Triton X-100 (100% degranulation); chimeric NIP IgE specific for the hapten 5-iodo-4-hydroxy-3-nitrophenyl (AbD Serotec) in the presence of absence of crosslinking stimulus (polyclonal antigen conjugated to Bovine Serum Albumin (NIP-BSA) or polyclonal rabbit α-human IgE (Dako)). Cells seeded at 1x10^4^ cells/well in culture medium overnight were sensitized with IgE (anti-NIP IgE, or anti-HER2 IgE, 200 ng/mL) or left in medium for 1 hour at 37°C, washed three times in HBSS buffer (Hank’s Buffered Salt Solution, 1% bovine serum albumin, Invitrogen) and stimulated at 37°C for 30 min with as appropriate. For β-hexosaminidase release detection, 50µl culture supernatants diluted 1:1 in HBSS buffer, plus 50 µl fluorogenic substrate per well (1 mM 4-methylumbelliferyl N-acetyl-β-D-glucosaminide, 0.1% dimethyl sulfoxide (DMSO), 0.1% Triton X-100, 200 mM Citrate, pH 4.5) were transferred onto black 96-well plate and incubated for 2 hours in the dark. Reactions were quenched with 0.5M Tris (100µl/well) and fluorescence was detected in a Fluostar® Omega microplate reader (350nm excitation, 450nm emission) (BMG Labtech). Degranulation was calculated as % of that measured with addition of Triton X-100 and compared with unstimulated cells (<10%).

**Basophil Activation Assay (BAT)**

The Basophil activation test (BAT) was carried out using the Flow2 CAST® kit (Bühlmann) according to the manufacturer’s instructions. Briefly, unfractionated human blood samples were stimulated, in the presence of stimulation buffer and anti-CCR3/anti-CD63 staining cocktail (Bühlmann), with anti-FcεRI, fMLP (Bühlmann) or anti-human IgE antibody (4.5 μg/ml final concentration; Dako) positive controls, or anti-HER2 or NIP IgE antibodies (3.5 μg/ml final concentration) for 30 minutes at 37°C^5^. Red blood cells were then lysed with 1x lysis solution (Bühlmann) for 10 minutes at room temperature. Samples were centrifuged and cells resuspended in acquisition buffer (Bühlmann). Activation of >500 CCR3^high^SSC^low^ basophils was assessed as up-regulation of CD63 expression by flow cytometry. Data is expressed as fold change in % CD63 expression upon stimulation, relative to the baseline level when incubated with stimulation buffer alone.

**Supplementary References**

1. Ilieva KM, Fazekas-Singer J, Achkova DY, et al. Functionally Active Fc Mutant Antibodies Recognizing Cancer Antigens Generated Rapidly at High Yields. Front Immunol. 2017;8:1112.
2. Dodev TS, Karagiannis P, Gilbert AE, et al. A tool kit for rapid cloning and expression of recombinant antibodies. Sci Rep. 2014;4:5885.
3. Bracher M, Gould HJ, Sutton BJ, Dombrowicz D, Karagiannis SN. [Three-colour flow cytometric method to measure antibody-dependent tumour cell killing by cytotoxicity and phagocytosis.](https://www.ncbi.nlm.nih.gov/pubmed/17531261) J Immunol Methods. 2007;323:160-71.
4. Karagiannis P, Singer J, Hunt J, et al. Characterisation of an engineered trastuzumab IgE antibody and effector cell mechanisms targeting HER2/neu-positive tumour cells. *Cancer Immunol Immunother.* 2009;58:915-930.
5. Rudman SM, Josephs DH, Cambrook H, et al. Harnessing engineered antibodies of the IgE class to combat malignancy: initial assessment of FcvarepsilonRI-mediated basophil activation by a tumour-specific IgE antibody to evaluate the risk of type I hypersensitivity. *Clin Exp Allergy.* 2011;41:1400-1413.

**Supplementary Table S1.** PIPE PCR primers

| PRIMER NAME | SEQUENCE 5’🡪3’ |
| --- | --- |
| F-1 | CGTACGGTGGCGGCGCCATCTGTCTTCATCTTCCCGCCAT |
| R-1 | GGAGTGCGCGCCTGTGGCGGCCGCCACCAAGAAGAGGATC |
| F-2 | GCTAGCACACAGAGCCCATCCGTCTTCCCCTTGACCCGCTGC |
| R-2 | ACCGCGGCTAGCTGGAACCCAGAGCAGCAGAAACCCAATG |
| F-HER-VH | GGCCGCCACAGGCGCGCACTCCGAGGTGCAGCTGGTGGAGTCT |
| R-HER-VH | ACGGATGGGCTCTGTGTGCTAGCTGAGGACACGGTCACCAGAG |
| F-HER-VL | CTGGGTTCCAGCTAGCCGCGGTGACATCCAGATGACCCAGTCT |
| R-HER-VL | AGATGGCGCCGCCACCGTACGTTTAATTTCAACTTTGGTACCTTGACC |


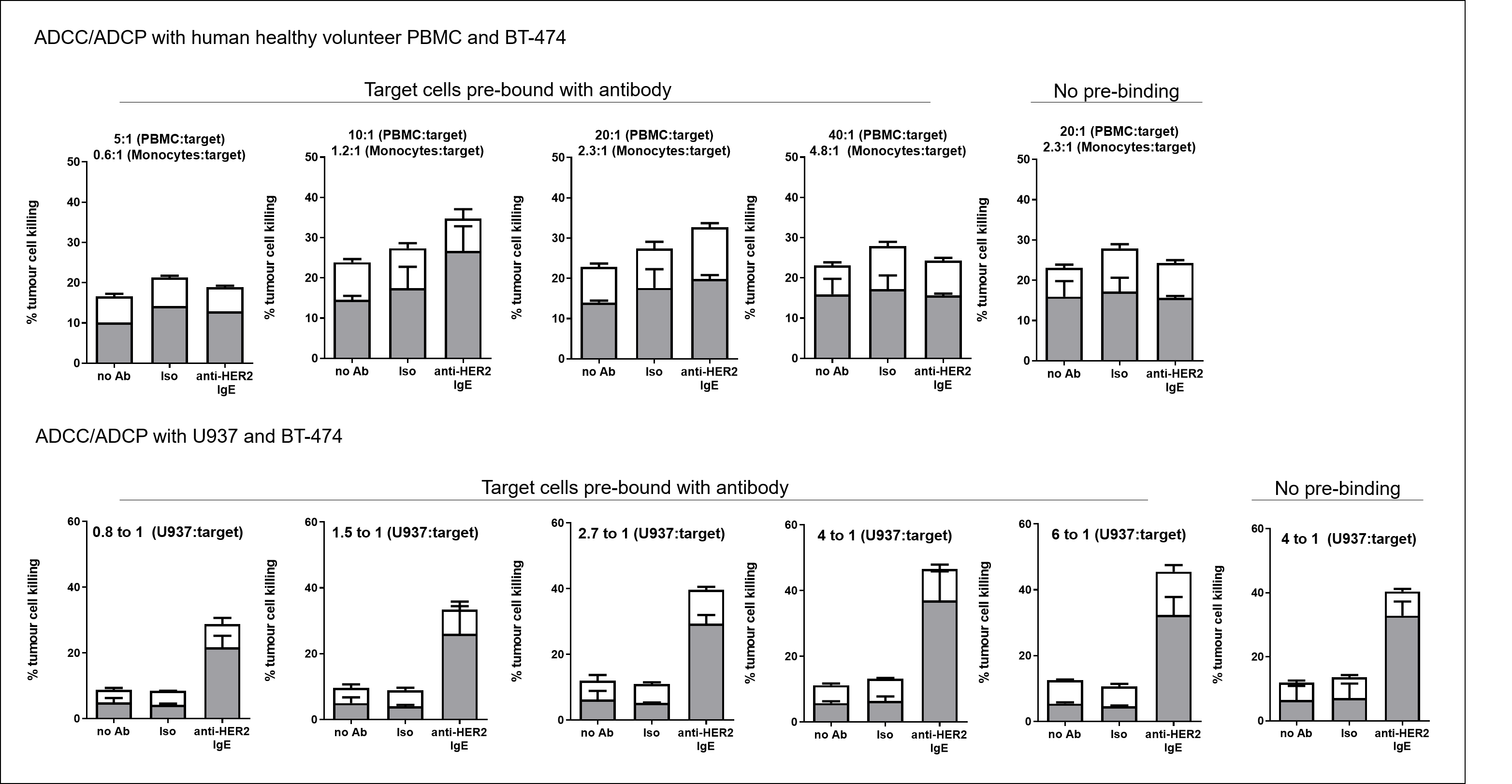


**Supplementary Figure S1. Optimisation of conditions for the ADCC/ADCP assay.** Optimal conditions were established for ADCC/ADCP assays with different effector cell populations: human PBMCs (monocyte effector:target ratios were calculated by flow cytometric analyses) (top panels) and with U937 monocytes (bottom panels). Conditions were established by evaluating different effector:target ratios, and by pre-binding of antibodies to tumour cells, compared to introducing antibody with effector and target cells.

**
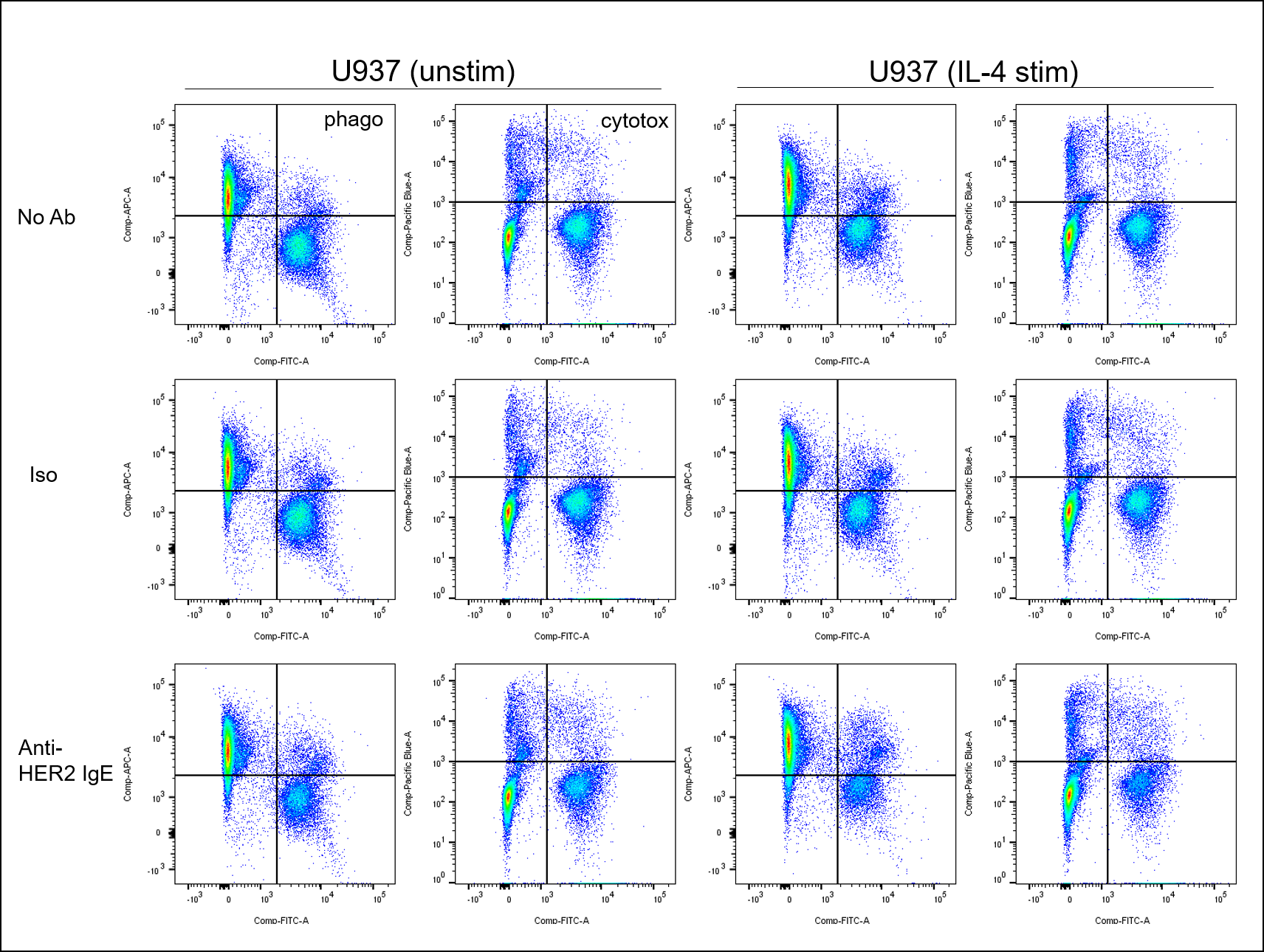
**

**Supplementary Figure S2. ADCC/ADCP assay representative dot plots.** Representative dual colour flow cytometric dot plots from which HER2 IgE-mediated anti-tumour phagocytosis (ADCP, left panels) cytotoxicity (ADCC, right panels) and measurements were conducted. In these examples, breast cancer cells were pre-stained with CFSE cell tracking dye (FITC-A), U937 monocytic cells were stained with anti-CD89 APC-conjugated antibody (APC-A), and dead cells were identified using DAPI live/dead dye (Pacific Blue-A). Left panel dot plots depict CFSE+ tumour cells (x-axis) and CD89-PE+ monocytic cells (x-axis) to quantitate total CFSE+ tumour cells and the number of tumour cells present within CD89+ monocytic cells, depicting ADCP by monocytic cells (CFSE+/PE+ cells). Right dot plots depict CFSE+ tumour cells and DAPI+ events (CFSE+/DAPI+ cells), allowing quantitation of tumour targets killed externally (ADCC). Mixed cell populations were incubated with no antibody (No ab), or with non-specific IgE isotype control (iso) or with anti-HER2 IgE. Incubation of HER2/*neu*-expressing breast cancer and U937 cells with anti-HER2 IgE was associated with increased tumour cell death by ADCC.
